# Recharging your fats: CHARMM36 parameters for neutral lipids triacylglycerol and diacylglycerol

**DOI:** 10.1016/j.bpr.2021.100034

**Published:** 2021-11-20

**Authors:** Pablo Campomanes, Janak Prabhu, Valeria Zoni, Stefano Vanni

**Affiliations:** 1 Chemin du Musée 10, Department of Biology, University of Fribourg, Fribourg, Switzerland

## Abstract

Neutral lipids (NLs) are an abundant class of cellular lipids. They are characterized by the total lack of charged chemical groups in their structure, and, as a consequence, they play a major role in intracellular lipid storage. NLs that carry a glycerol backbone, such as triacylglycerols (TGs) and diacylglycerols (DGs), are also involved in the biosynthetic pathway of cellular phospholipids, and they have recently been the subject of numerous structural investigations by means of atomistic molecular dynamics simulations. However, conflicting results on the physicochemical behavior of NLs were observed depending on the nature of the atomistic force field used. Here, we show that current phospholipid-derived CHARMM36 parameters for DGs and TGs cannot adequately reproduce interfacial properties of these NLs because of excessive hydrophilicity at the glycerol-ester region. By following a CHARMM36-consistent parameterization strategy, we develop improved parameters for both TGs and DGs that are compatible with both cutoff-based and particle mesh Ewald schemes for the treatment of Lennard-Jones interactions. We show that our improved parameters can reproduce interfacial properties of NLs and their behavior in more complex lipid assemblies. We discuss the implications of our findings in the context of intracellular lipid storage and NLs’ cellular activity.

## Why it matters

Neutral lipids play a key role in the field of lipid metabolism, most notably lipid transport and accumulation. Currently available force-field parameters to investigate the dynamics of these lipids in silico have major shortcomings, and their usage has led to erroneous interpretations of their behavior in complex lipid assemblies that mimic cellular membranes. In this work, we developed improved CHARMM36-consistent parameters for diacylglycerols and triacylglycerols that allow us to accurately reproduce lipid interfacial properties and acquire structural information in complex lipid assemblies to complement experimental efforts in a global understanding of the behavior of cellular lipids both in vitro and in vivo.

## Introduction

Neutral lipids (NLs), generally defined as naturally occurring hydrophobic molecules that lack any charged chemical group, are an important class of cellular lipids (1). As a consequence of their extreme hydrophobic character, they are immiscible in water, and above their transition temperature, they do not self-assemble in lamellar phases such as lipid bilayers, but rather behave as pure liquids. They are thus characterized by classical properties of liquids, including density, surface tension (ST) with the gas phase, and interfacial tension (IT) with water.

Among NLs, most of the attention has focused on cholesterol, both because of its abundance in the cell and its implications in countless cellular processes and diseases. As a consequence, cholesterol is also the most studied NL from a structural perspective. To this extent, computational investigations of cholesterol using molecular dynamics (MD) simulations, the method of choice to investigate lipid assemblies at the structural level (2,3), date back to the 90s (4, 5, 6).

Recently, however, other NLs involved in phospholipid synthesis and degradation, most notably triacylglycerol (TG) and diacylglycerol (DG), have received increasing attention at the structural level (7, 8, 9, 10, 11, 12, 13, 14, 15, 16, 17, 18), in large part because of ever-increasing focus on cellular lipid storage and related metabolic diseases (19). To this extent, both fully atomistic (all-atom) and coarse-grained (CG) MD simulations of TG- and DG-containing systems have been reported using different force fields (7, 8, 9, 10, 11, 12,20, 21, 22, 23, 24, 25, 26, 27).

Historically, the first all-atom parameters for NLs were prepared based on the OPLS-compatible Berger parameterization strategy (12), and they have been successfully used to investigate several structural aspects of high-density and low-density lipoproteins (11,18), lipid-droplet-like surfaces (20), and oil-water interfaces (17). More recently, new parameters compatible with the widely successful CHARMM36 (C36) force fields for lipids have been used to investigate the properties of lipid assemblies containing TGs, in the absence or presence of accompanying proteins (22,23,27). These parameters, C36-s (C36-standard) from now on, are derived from those developed for phosphatidyl-choline (PC) lipids by simply replacing the charged phosphate and choline groups with one additional acyl chain in position *sn*-3.

Quite remarkably, however, the two parameter sets (Berger versus C36-s) produce contrasting results on the key physicochemical properties of TG assemblies. Most notably, the two models suggest very different hydration properties for TG, with the C36-s model showing ∼10 times more water molecules in bulk liquid TG (22) with respect to simulations carried out with Berger parameters (20). This is even more confounding when considering that the reported IT of trioleoylglycerol (triolein, TOG) with water is very similar (31 ± 2 mN/m for Berger (11), 29.7 ± 1.7 mN/m for C36-s (22)) for the two models and, in both cases, close to the reported experimental values (29.2 (28) and 32 mN/m (29)).

From a biological perspective, the simulations carried out with the more hydrophilic C36-s set of parameters have led to a model in which TG molecules (named “SURF-TG”) can reside at the surface of lipid bilayers, adopting phospholipid-like conformations and acting as monolayer lipids (22). This interpretation has potentially profound implications for what pertains to both integral and peripheral proteins interactions with lipid droplets, as well as for the mechanism of TG degradation by lipases (23,25).

Here, we investigated the origin of the aforementioned discrepancies in simulations carried out with the two TG models. We encountered significant shortcomings in the current C36 force field parameters for TG and found that a similar criticality was present in another NL (DG). To solve this issue, we developed improved C36-compatible parameters for both TG and DG, which were validated against experimentally reported key physicochemical properties of these NLs.

## Materials and methods

### Systems setup

Suitable starting configurations to compute DOG (1,2-dioleoyl-*sn*-glycerol) and TOG ITs were obtained by backmapping CG equilibrated snapshots. These snapshots were extracted from CG MD simulations that used force fields for TOG and DOG developed in previous studies (9,20). These “oil in water” structures contained an oil core composed of either 512 DOG or 432 TOG molecules immersed in water and led to equilibrated periodic boxes with sizes of ∼85 × 85 × 125 Å^3^ and 105 × 105 × 120 Å^3^, respectively.

The solvated TOG equilibrated structure, upon removal of the water molecules, was also used to initiate the simulations required to compute the oil density. On the other hand, starting random configurations to calculate density and ST for other molecules in the training set (see Table 1) were built using PACKMOL (30). Every system was composed by 512 identical molecules.

**Table 1:** Training target data

System	Property	Mean value	95% CI	Scaling factor
TOG	IT	30.6	2.4	0.040
DOG	IT	17.2	1.4	0.040
EOOP	ST	23.8	1.9	0.035
EGDA	ST	33.0	2.6	0.035
TAGL	ST	37.0	3.0	0.035
EOOP	density	0.884	0.018	1.000
EGDA	density	1.101	0.022	1.000
TAGL	density	1.158	0.023	1.000
TOG	density	0.909	0.018	1.000

Data are presented as mean values and estimated 95% CIs. Densities are given in g/cm^3^, and STs at the oil/air interface and ITs with water are given in mN/m. The scaling factors used to make sure that all training targets had a similar order of magnitude during the iterative optimization protocol are also shown. The chemical structures of all systems are displayed in Fig. 3*A*. CI, confidence interval; EOOP, ethyl propionate; EGDA, ethylene glycol diacetate; TAGL, triacetylglycerol.

The systems used to estimate the DOG and TOG flip-flop rates in palmitoyl-oleyl (PO)PC bilayers were built according to the following procedure. We prepared four replicas for each of the systems (POPC/DOG and POPC/TOG). Two of them were fully generated using CHARMM-graphical user interface (GUI) (31,32) by randomly inserting an equal amount of oil molecules (DOG or TOG) in each of the POPC leaflets. For the other two, we used CHARMM-GUI to initially build pure POPC systems; their corresponding leaflets were then separated along the *z* direction (perpendicular to the bilayer-water interface) to sandwich a layer of NLs (DOG or TOG) in between. All the POPC/oil systems were composed of 800 lipids. The molar concentrations of lipids were 96:4 for POPC/TOG and 90:10 POPC/DOG, and the lipid/water ratio of all the systems was set to 1:50.

### Parameterization protocol

**Figure 1:**
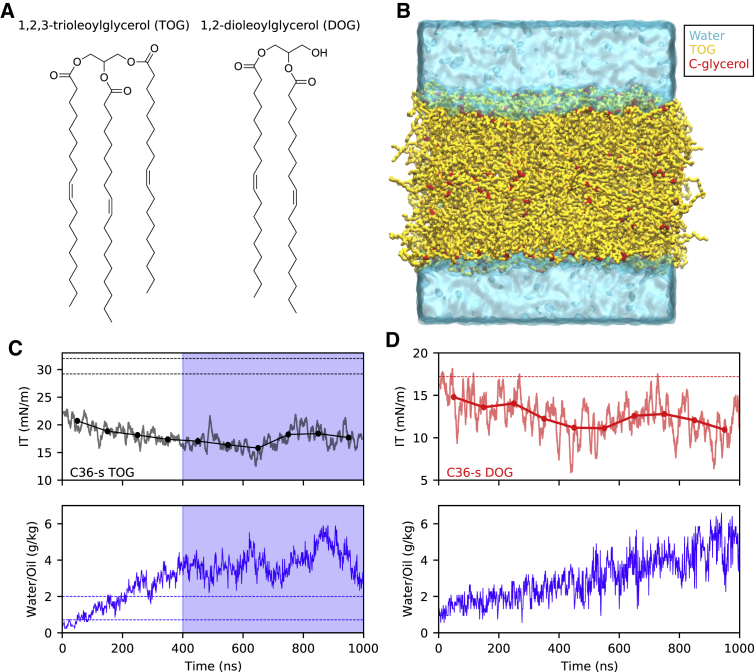
IT of TOG and DOG computed from simulations carried out using the C36-s model. (*A*) Chemical structures of TOG and DOG. (*B*) Setup for IT calculations. Water is represented in cyan, TOG in yellow, and the carbons of the glycerol group (C1, C2, C3) in red. (*C*) IT values for TOG as running averages (*solid lines*) and blocking averages (mean values every 100 ns) (*top*) and water content inside the oil core (*bottom*). The horizontal dashed lines represent the experimental values reported for both IT (*top*, *black*) and water content (*bottom*, *blue*). The blue shaded region contains the part of the trajectory used to calculate the mean IT value for TOG, when water content shows convergence. (*D*) IT values for DOG as running averages (*solid lines*) and blocking averages (mean values every 100 ns) (*top*) and water content inside the oil core (*bottom*). The horizontal dashed line represents the experimental value reported for DOG IT (*top*, *red*).

To develop new parameters for TOG and DOG, our working hypothesis was that the topologies commonly used for these molecules, taken from standard repositories (22,31), present atomic point charges on their ester and glycerol groups that are inaccurate, and that is the sole factor responsible for an excessive hydration near this region (see Fig. 1). Therefore, to maximize the compatibility with the existent C36 force fields for phospholipids (33,34), we decided to employ a minimal parameterization strategy, according to which only the atomic charges on the ester-glycerol skeleton of TOG and DOG were optimized, whereas the rest of the nonbonded parameters, particularly the charges on the acyl chains, and all bonded parameters were kept at their original values. To keep compatibility with the existent C36 schemes to treat the Lennard-Jones (LJ) interparticle interactions and cutoff-based and particle mesh Ewald (PME) approaches, we developed two different set of parameters: C36-cutoff (C36-c) and C36-PME (C36-p), respectively. Their development followed a workflow similar to that previously employed to generate optimal parameters for phospholipids within the C36-LJ-PME framework (34). As described below in further detail, besides a different treatment of the LJ interactions, some other distinct choices were taken to build these two models. Concretely, different sets of initial charges and of training targets were employed.

All classical MD simulations in this study were performed under periodic boundary conditions using the version 2020.4 of GROMACS (35, 36, 37) and shared some common settings: 1) the long-range electrostatic atomic interactions were taken into account by means of the PME algorithm (38) with a Fourier grid space of 0.12 nm and a real-space cutoff of 1.2 nm; 2) all bonds involving hydrogen atoms were constrained using the Linear Constraint Solver (LINCS) and SETTLE algorithms (39,40), thus allowing the usage of an integration time step of 2 fs; and 3) the TIP3P model (41) was employed to describe water molecules in all lipid-water simulations. Besides the number of particles and the temperature, either the pressure or the volume was controlled in all simulations. Pressure control varied depending on the property to be computed from the simulations: 1) isotropic (in all dimensions) for densities and 2) only in one dimension (*xy* constrained to the original values, *z* allowed to fluctuate) for IT. On the other hand, no pressure control was applied in the simulations performed to calculate ST; the canonical (NVT) ensemble was used in this scenario.

### C36-c model

To get an initial set of atomic charges (fine-tuned at a later stage) for this model, we first sampled the conformational space of a TOG molecule in vacuum by means of an 80-ps-long MD run. From this simulation, which was performed with a semiempirical method (Austin Model 1, AM1) (42), we extracted 200 frames, and for each of them we performed a single point energy calculation at the Hartree-Fock (HF)/6-31G(d) theory level (43) using the conductor-like polarizable continuum model (44) to implicitly mimic a water environment. All these electronic structure calculations were performed using the ORCA package (45). This protocol allowed us to obtain conformational energies and wave functions at a theory level higher than AM1 while taking into account the effect of water interactions in the determination of the atomic charges, as dictated by CHARMM parameterization philosophy. The abovementioned wave functions were used to compute Charge Model 5 (CM5) atomic charges (46) for every configuration extracted from the dynamics via a subsequent population analysis, and lastly, these charges were then averaged to get the initial set to be optimized.

The optimization of these CM5 charges was performed using a gradient-based iterative procedure similar to that previously described (34). The gradient on the hypersurface defined by these parameters (atomic charges) was estimated via thermodynamic reweighting (47). To carry out this optimization and obtain a set of charges compatible with the glycerol-ester groups present in both TGs and DGs, DOG and TOG ITs were used as target properties. We estimated a mean value and a 95% confidence interval for TOG IT from the three independent experimental studies published in the literature (28,29,48). An analogous relative uncertainty (8%) was used to estimate the 95% confidence interval for DOG. These confidence intervals were employed to define the weights for the training targets; we define them as inversely proportional to their corresponding uncertainties. A regularization strategy that restrained the final set of atomic charges to stay as close as possible to the original CM5 set was applied to avoid overfitting. To this end, we used regularization weights that ensured a maximal change of 0.005e on any partial charge during the optimization cycles. At the end of every optimization cycle, mean values and confidence intervals were extracted from the distributions sampled during the dynamics. The iterative protocol was run until enough overlap was observed between the corresponding confidence intervals (experimental data versus simulations).

In all MD simulations required for the parameterization, constant temperature (298.15 K) and pressure (1 atm) were imposed by coupling the system to a stochastic velocity rescaling thermostat (49) with a coupling time constant of 1 ps and a Parrinello-Rahman barostat (50) with a coupling time constant of 5 ps, respectively. The van der Waals interactions were truncated using a cutoff value of 1.2 nm and a standard smoothing function for the tail region (1.0–1.2 nm). Because of the correlation observed in our simulations between water content inside the oil core and IT, we monitored water penetration inside the oil core along the dynamics and used it as a measure of equilibration (see Figs. 5 and S2). After relatively long equilibration periods, production runs of at least 500 ns were carried out to collect enough statistics and get accurate estimates for the IT of the different oils (DOG and TOG). The surface tensions were computed from the diagonal values of the pressure tensor (*P*_*xx*_, *P*_*yy*_, and *P*_*zz*_) using the Kirkwood-Irving method (51), as follows:

(1) $$\gamma =\frac{L}{2}\left\langle P_{zz}-\frac{P_{xx}+P_{yy}}{2}\right\rangle ,$$

where *L* represents the box length along *z* and <…> denotes ensemble average.

### C36-p model

To build this model, the C36-c optimal set of atomic charges was employed to initiate the optimization protocol. These charges were fine-tuned according to a procedure similar to that described above for the C36-c model (i.e., via thermodynamic reweighting), but in this case, the training set was expanded and various properties of compounds containing the glycerol-ester group in their molecular skeleton were selected as training targets (Table 1). To estimate confidence intervals for the experimental densities, we took the experimental data reported in the literature for TOG (52,53) and acted as described above in the case of IT. For STs, we used relative uncertainties of 8% in analogy to those employed for IT experimental values (Table 1). The weights for the training targets had to be scaled in this case because the order of magnitude of the training targets was quite different. The scaling factors used to solve this issue are collected in Table 1. As before, regularization weights were chosen to control the change on the atomic charges and keep them as close as possible to the original set. In this case, the regularization weights were selected to allow a maximal change of 0.02e on any partial charge at every optimization step. At the end of every optimization cycle, mean values and confidence intervals were extracted from the distributions sampled during the dynamics. The iterative protocol was run until enough overlap was observed between the corresponding confidence intervals (experimental data versus simulations). The optimal set of atomic charges for this C36-p model is shown in Fig. S1.

Constant temperature and pressure (when applicable) were imposed using the same algorithms and parameters described in the previous section (C36-c model). After equilibration (determined, in the case of IT, by a constant average value of the water content inside the oil core), production runs of 25 ns, 100 ns, and not shorter than 500 ns were carried out to calculate density, ST, and IT, respectively. ST and IT were obtained using Eq. 1 above. In all MD simulations required to build the C36-p model, a real-space cutoff of 1.0 nm was employed to treat the van der Waals interactions at short range, whereas the recently developed LJ-PME algorithm (54) was used to treat all interparticle interactions at larger distances (long range).

### Flip-flop energy barriers for DOG and TOG

All MD simulations carried out to investigate POPC-oil-water systems were performed in the isothermal-isobaric (NPT) ensemble using semi-isotropic pressure control and analogous settings to those described above for the cutoff and PME-like treatment of the LJ interactions in C36. The lateral density profiles for the phosphorus atom type (P) of POPC and carbon (C2) of the ester group of the oils (DOG and TOG) were calculated using the *gromacs density* tool. The bin size for calculating these profiles was set to 0.07 nm. The potentials of mean force (PMFs) were computed by Boltzmann inverting the corresponding density profiles, using the C2 atom as reference. The simulations were carried out for 400–800 ns, until the estimated energy barriers converged. The final part of the trajectories for the different replicas (200–300 ns) was then used for analyses. The final values for the PMFs are reported as average and standard deviation over four replicas.

### Data availability

All input files used to run the MD simulations and data required to reproduce the figures in this manuscript are available on Zenodo (https://zenodo.org/record/5599949#.YZe_4udOnIU).

## Results

To investigate the incongruity between the two parameter sets and because the results with the Berger model have been validated by a number of independent research groups (11,17,20), we opted to start by computing the IT of TOG via MD simulations on a flat TOG-water interface (Fig. 1, *A* and *B*) using the C36-s parameters (see Materials and methods for further details). As can be appreciated in Fig. 1, the IT estimated from the analysis of the trajectory obtained when the C36-s model was employed for TOG diverges from the expected range of experimental measurements (29–32 mN/m), instead plateauing to a value (unless otherwise stated, from now on, uncertainties will be reported as 95% confidence intervals) of 17.3 ± 2.0 mN/m after a relatively long equilibration period (Fig. 1 *C*). Of note, a similar discrepancy between the experimental IT value and that obtained with the C36-s model has been reported in a recent manuscript (21). Interestingly, we observed that this decrease in IT is coupled to a linear increase in the hydration of the TOG core (Fig. 1 *C*), which could explain the long simulation time required to equilibrate this system (∼400 ns). At this stage, the amount of water that penetrates into the oil core stabilizes to some extent, and the IT profile displays the expected convergence fingerprint, which allows estimating an IT average value for this system. This observation provides a likely explanation for the difference between our result (17.3 mN/m) and that previously reported (22) (29.7 mN/m), as in that study, the IT for TOG was computed from a short 50 ns run.

We next investigated whether a similar criticality was present in another NL, DOG. DOG is the natural precursor of both TOG and cellular phospholipids, and, unlike TOG, is generally considered as a bona fide bilayer lipid, even though its presence above 20–30% molar concentration in phospholipid bilayers promotes the formation of nonlamellar structures (55). Indeed, for DOG we observed a similar behavior to that of TOG (Fig. 1 *D*); in this case, even if the simulation seems to still lack convergence because water is still penetrating into the oil core after a long 1 *μ*s run, the IT at the DOG water interface calculated using the C36-s model significantly deviates from that experimentally measured (17.2 mN/m (56)).

To solve this issue, and especially considering that the C36 force field is widely used in MD simulations of lipid systems, we next investigated the origin of the inability of the C36-s model to reproduce this key experimental parameter. We noticed that during the development of the C36 parameters for lipids, the charges on the ester groups of PC lipids, from which those on TG and DG molecules derive, were explicitly increased with respect to the Quantum Mechanical (QM)-derived charges to reproduce the correct hydration of PC lipids (33). Thus, to minimally affect C36 parameters that have shown very good agreement with experimental data (33), we opted to reparameterize exclusively the charges on the glycerol and ester groups using C36-compatible strategies (33,57). Those present in the acyl chains and the alcohol group (in the case of DOG), as well as the rest of nonbonded parameters and all bonded parameters, were not altered and therefore kept at their original C36 values.

**Figure 2:**
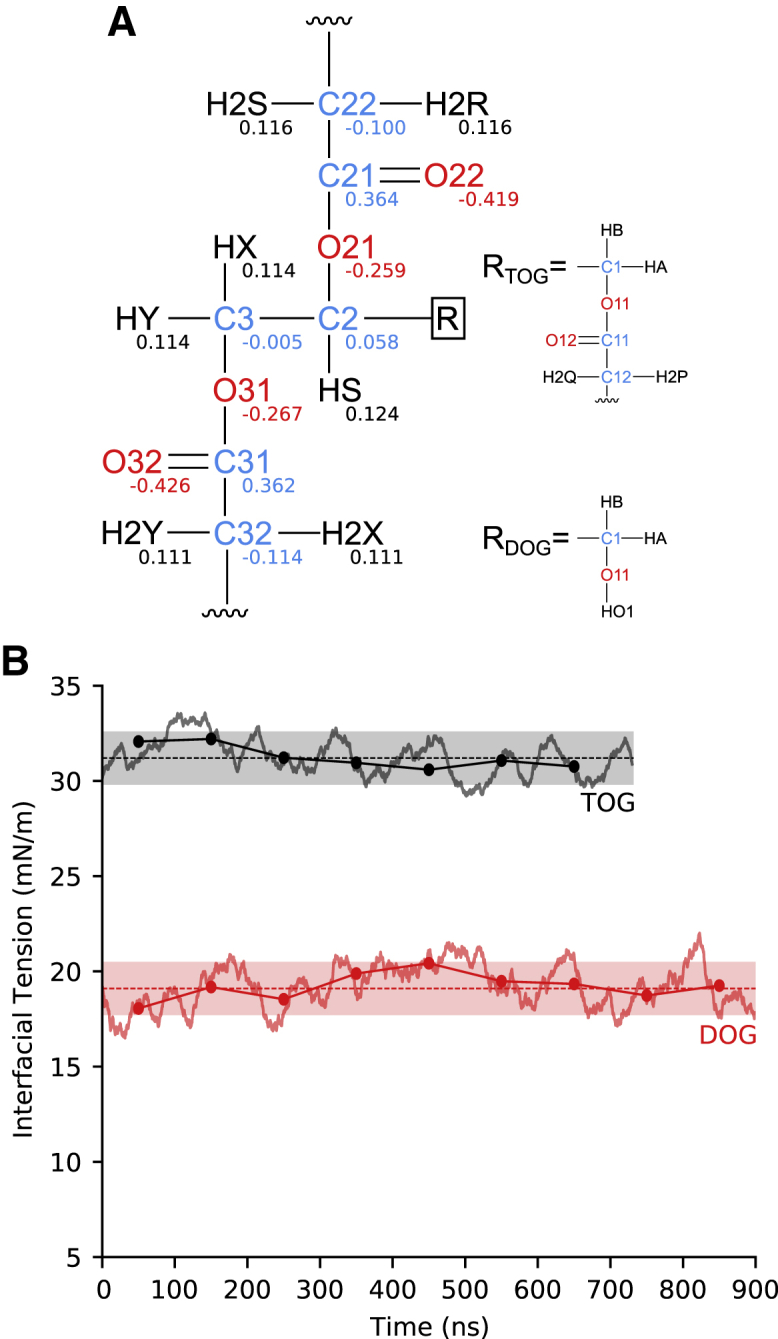
IT of TOG and DOG computed from simulations carried out using the C36-c model. (*A*) Fine-tuned set of charges for TOG and DOG. For TOG, the charges of atoms in chain 1 are identical to those shown for chain 3. (*B*) IT values for TOG and DOG as running averages (*solid lines*) and blocking averages (mean values every 100 ns) (*dotted solid lines*). Only the equilibrated part of the trajectories is shown. Mean values (*dashed horizontal lines*) and their corresponding 95% confidence intervals extracted from the simulations are also displayed.

In short, we initially got a conformationally consistent set of CM5 atomic charges (46) for TOG that, as dictated by CHARMM parameterization philosophy, was determined from a population analysis of quantum wave functions determined by implicitly taking into account water-TOG interactions. Then, this initial set of charges was fine-tuned following a regularized gradient-based iterative procedure similar to that previously outlined (34) (see Materials and methods for further details on the parameterization protocol). With the improved parameters, obtained after running two cycles of the above-described iterative procedure (Fig. 2 *A*), the IT values for TOG and DOG converged to 31.2 ± 1.4 and 19.1 ± 1.4 mN/m, respectively (Fig. 2 *B*). These are in close agreement with those experimentally measured. It must be remarked that this optimal set of parameters, C36-c, was developed considering a cutoff-based treatment of the LJ interactions and can therefore be used in combination with the existent 12 Å cutoff C36 force fields for proteins (58,59) and phospholipids (33,34).

**Figure 3:**
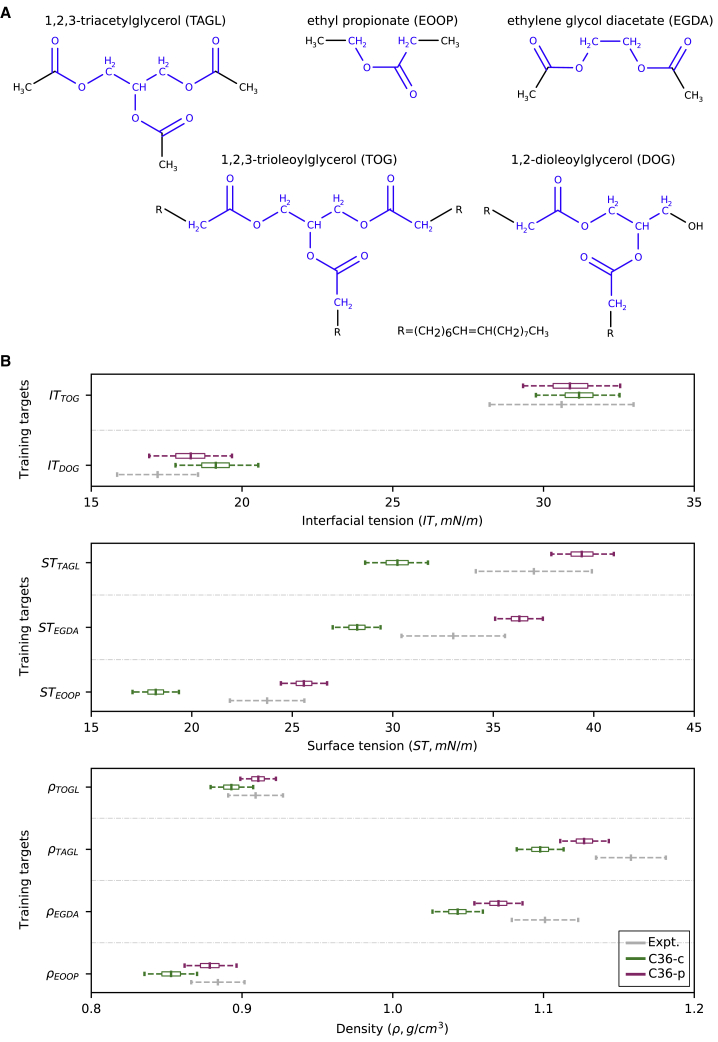
Parameterization approach for C36 force field using LJ-PME (C36-p). (*A*) Set of molecules used as training set to carry out the C36-p parameterization. The atoms for which charges were optimized are colored in blue. (*B*) Densities (*ρ*, in g/cm^3^), surface tensions (STs, in mN/m), and ITs (in mN/m) experimentally measured (*gray*) and computed using the C36-c (*green*) and C36-p (*magenta*) sets of parameters. Mean values and 95% confidence intervals are shown. The boxes represent the first and third quartiles of the distributions sampled during the dynamics. Confidence intervals for the experimental measurements were estimated as described in Materials and methods.

We next decided to assess the ability of the C36-c model to reproduce other experimental properties in molecules containing the same glycerol-ester skeleton found in TGs and DGs. To this end, we investigated whether, using the C36-c set of parameters, it was possible to accurately estimate TOG density, and the density and ST of a set of molecules (Fig. 3 *A*) for which these two properties have been experimentally measured (60). Of note, although densities are in relatively good agreement with the experimentally reported values (showing relative errors below 5%), STs are not well-reproduced by the C36-c model and present relative errors in the 15–23% range. However, this is a well-known issue of the C36 lipid model (34,61) that, as shown for several alkanes and oils (54), originates from the cutoff-based treatment of LJ interactions.

Fortunately, a solution to this problem has been recently proposed via the use of a PME-like treatment of the LJ interactions in C36 (LJ-PME) (34,61). We thus opted to also develop C36 LJ-PME-compatible parameters for TGs and DGs. To do so, we used a thermodynamic reweighting procedure similar to that described above, but targeting not only TOG and DOG IT but also TOG density, as well as the density and ST of the molecules displayed in Fig. 3 *A*. By doing so, we also expect a larger model transferability. We used the optimal set of charges of the C36-c model to initiate the protocol, which was then fine-tuned as described above but now activating the usage of LJ-PME in our simulations. After a single iteration, we obtained a new set of charges, C36-p (Fig. S1), that was able to reproduce fairly well all the properties targeted (Fig. 3 *B*). In particular, the mean relative errors for densities and ST were ∼1 and 8%, respectively. Moreover, as shown in Fig. 3 *B*, this new set of charges jointly led to TOG and DOG IT values (30.9 ± 1.6 and 18.3 ± 1.4 mN/m, respectively) that are in very good agreement with the reported experimental measurements.

**Figure 4:**
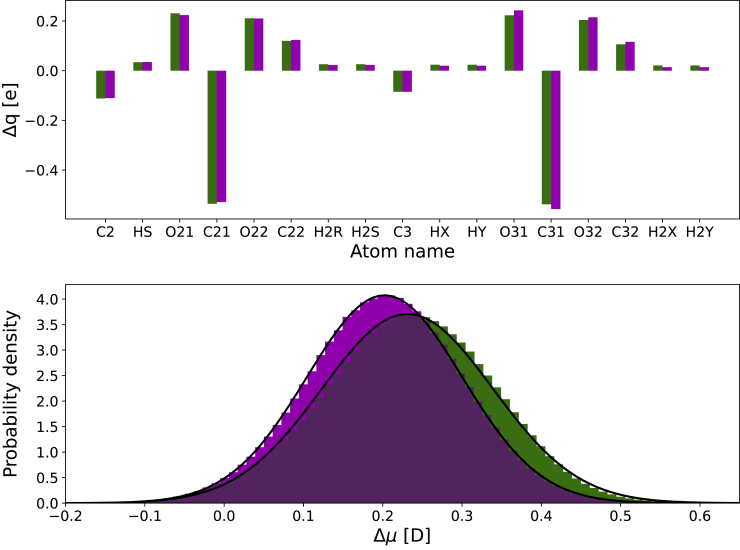
Differences in partial charges and dipole moment of C36-c and C36-p models with respect to standard C36-s parameters. (*Top*) Change in partial charges (Δq) of the atoms belonging to the glycerol-ester region. The variation in the atomic charges of the C36-c and C36-p parameterizations relative to those in the C36-s model are shown in green and magenta, respectively. (*Bottom*) Change in the dipole moment (Δ*μ*) created by the glycerol-ester region. The differences between the dipole moments computed when using the C36-c and C36-p parameterizations with respect to those obtained with the C36-s model are shown as probability density distributions in green and magenta, respectively. A bin width of 0.01 D was used to build the histograms. Gaussian fits to the histograms are also displayed.

The changes in the partial charges of the atoms belonging to the glycerol-ester region with respect to those in the C36-s model are shown in Fig. 4. As illustrated there (*top panel*), the charges located on the carbonyl carbons are those that experience the largest adjustments. Their positive charges are noticeably decreased in relation to those found in the C36-s parameterization. Interestingly, this effect is partially compensated by an opposite change in the charges of the oxygen atoms bound to them, which globally makes the bonds between the atoms in this region less polar in the C36-c and C36-p models. Of note, atomic charges are only slightly tuned when moving from the C36-c to the C36-p parameterization.

The modification on the partial charges of the atoms in the glycerol and ester groups translates into a change in the related dipole moment of this molecular fragment. To investigate to what extent these distinct atomic charges impact the dipole moment created by the glycerol-ester region, we first built a glycerol-ester conformational ensemble by extracting the coordinates of every glycerol-ester moiety (three per TOG molecule) for all TOG molecular conformations sampled along the simulations. Then, for each structure of this common ensemble, we computed the change in the modulus of the dipole moment (compared to that obtained with C36-s) when the C36-c and C36-p sets of charges were used. The corresponding distributions are displayed in Fig. 4 (*bottom panel*) and show that the dipole moments calculated using the new sets of charges are slightly larger on average: 0.23 and 0.20 D when using the C36-c and C36-p parameterizations, respectively. Notably, the orientation of the dipole moment is barely altered (see Fig. S3).

**Figure 5:**
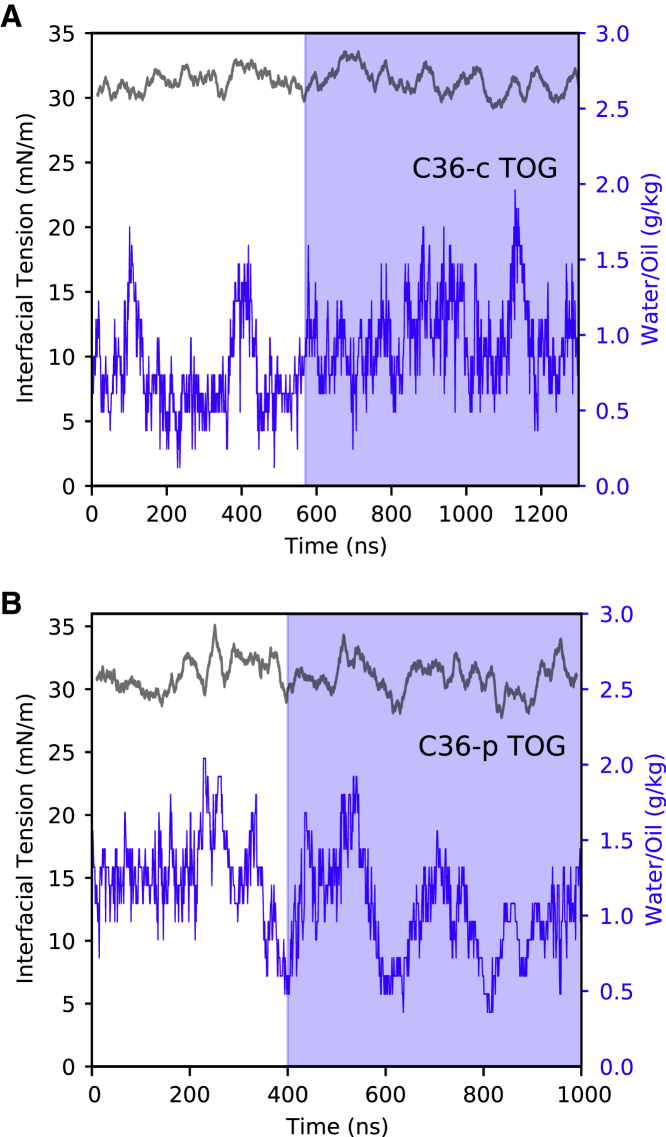
IT (*black*) and water content (*blue*) of TOG computed from simulations carried out with the improved parameters C36-c and C36-p. (*A*) Results from the C36-c model. (*B*) Results from the C36-p model. The blue shaded region contains the part of the trajectory used to calculate the mean IT value for TOG, when water content shows convergence.

Next, as an independent additional validation, we estimated the amount of water dispersed in the oil core in the case of TOG (for which this property has been experimentally measured (62)) from the simulations performed with the C36-c and C36-p models (Fig. 5). As mentioned above, it appears that there is a correlation between the IT values and the water content inside the oil core. This correlation is not unexpected, as both properties are related to the hydrophilicity of the oil molecules. In particular, when the C36-c and C36-p models were used, mean values of 0.98 and 1.01 g/kg water/oil, respectively, were found for the water-holding capacity of TOG. These computed values are in good agreement with the experimentally reported range of 0.8–2 g/kg water/oil for TG samples (62), thus giving some initial support to the adequacy and improved accuracy of the parameter sets developed in this study.

**Figure 6:**
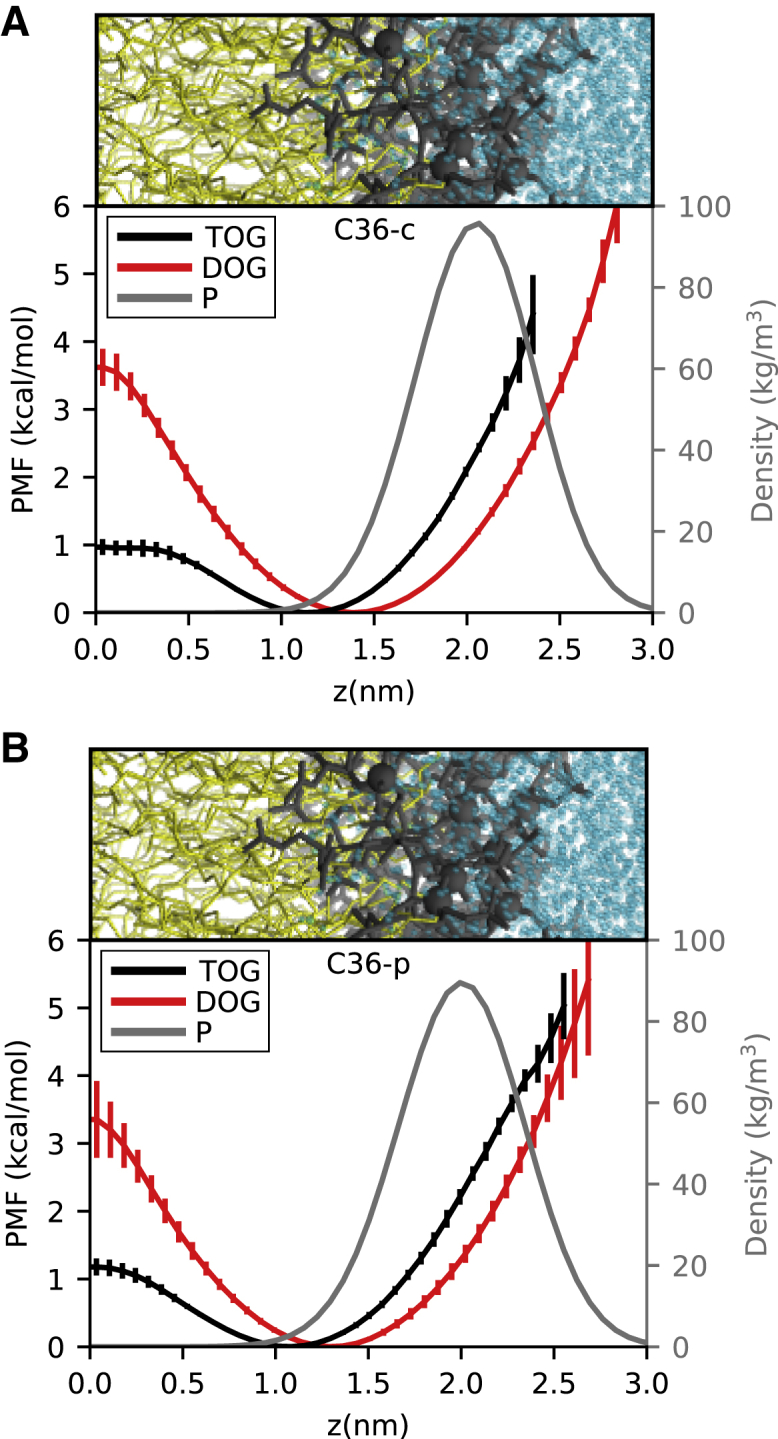
PMFs calculated for TOG (*black*) and DOG (*red*) in POPC bilayers. (*A*) Results for simulations obtained using the improved C36-c parameters for TOG and DOG. (*B*) Results from simulations with the improved C36-p parameters for TOG and DOG. The PMF curves are obtained as an average over four replicas. The associated uncertainties (standard deviations) are also shown together with the estimated density profile for the phosphate group (P) of POPC (*gray line*), which was computed as an average between both TOG/POPC and DOG/POPC simulations.

Finally, to further validate the accuracy of the parameter sets developed in this work (C36-c and C36-p), we investigated the behavior of DOG and TOG in the presence of bilayer-forming lipids, as this is the most common setup for the study of physiologically relevant properties of NLs. To do so, we estimated the flip-flop energy barrier of physiologically abundant monounsaturated TG and DG molecules (TOG and DOG) in POPC lipid bilayers using either C36-c or C36-p for the NLs in combination with the corresponding CHARMM force fields (C36 or C36-LJ-PME, respectively) for the phospholipids (Fig. 6). The PMFs shown in Fig. 6 were computed using Boltzmann inversion from four independent MD simulations using different initial configurations for the various replicas (see Materials and methods for further details). The obtained average values for the flip-flop energy barriers amount to ∼1 kcal/mol for TOG and 3.5 kcal/mol for DOG. The latter is in good agreement with that experimentally estimated for DG molecules (63), which further supports the strategy employed for the parameterization and the accuracy of the charges here developed for the glycerol-ester groups.

## Discussion

In this work, we developed improved C36-compatible parameters for two NLs, TG and DG, and we validated our new parameters against experimentally reported physicochemical properties of these NLs. Most notably, our results allow to evaluate the potential biological implications of a (in)correct treatment of the hydrophobic-hydrophilic balance in MD simulations of NLs. Our data indicate that the glycerol moiety of TG molecules is most abundant in the hydrophobic interior of the bilayer, albeit not at the interface between the two monolayers, but rather 1 nm above it (Fig. 6). This confirms previous models suggesting that the presence of TG molecules in bilayers can alter the arrangement of bilayer phospholipids (20,22,25). On the other hand, the energetic cost to move from one monolayer to the other is very low (1 kcal/mol) and significantly lower than the cost of reaching the bilayer surface (∼2.5 kcal/mol), defined as the average level of the phosphate groups of the phospholipids (Fig. 6). Hence, TG flip-flop between monolayers is extremely fast, thus excluding potential monolayer activity of TG molecules that could lead to any sort of TG-induced asymmetric behavior, such as membrane bending (21) or lipid droplet budding (13), in the presence of symmetric lipid bilayers.

DG, on the other hand, displays a behavior that is intermediate between that of TG and that of phospholipids (64) (Fig. 6). As such, it should not be simply thought of as a “classical” glycerolipid, with its polar head pointing toward water and its acyl chains embedded in the hydrophobic core of the lipid bilayer, but rather as a more complex, multifaceted molecule with a physicochemical complexity that mirrors its biological one (9).

In conclusion, we identified significant shortcomings in the current C36 force field parameters for TG and DG molecules. These issues are the origin of reported discrepancies in the description of oil-water and oil-phospholipid-water interfaces using MD simulations. Here, we provide improved parameters compatible with the C36 force fields for proteins and phospholipids based on a minimal parameterization strategy that focuses on reducing the excessively high point charges on the ester and glycerol groups of the molecules. Our improved models provide excellent agreement with key physicochemical properties of NLs, and they are fully compatible with cutoff-based and PME schemes for the treatment of LJ interactions. We foresee that the parameters developed in this work will be of help to address the ever-increasing interest in the structural role of NLs in modulating key physiological processes related to cell homeostasis and related metabolic diseases.

## Author contributions

P.C. and S.V. conceptualized and designed the research. P.C., J.P., and V.Z. performed the simulations and data analyses. S.V. supervised the project; the manuscript was written through contributions of all authors.

## Declaration of interests

The authors declare no competing interests.

## Supporting material

Document S1. Figs. S1–S3

Document S2. Article plus supporting material
