# Supplementary material for: Recharging your fats: CHARMM36 parameters for neutral lipids triacylglycerol and diacylglycerol
Source: Biophys Rep (N Y). 2021 Dec 8;1(2):None. doi: 10.1016/j.bpr.2021.100034 (PMC8651513; doi:10.1016/j.bpr.2021.100034)
Supplement: Supplementary file 1 [file mmc1.pdf]

Biophysical Reports, Volume *1*

Supplemental information

Recharging your fats: CHARMM36 parameters for neutral lipids triacyl-glycerol and diacylglycerol

Pablo Campomanes, Janak Prabhu, Valeria Zoni, and Stefano Vanni

Supplemental Figures

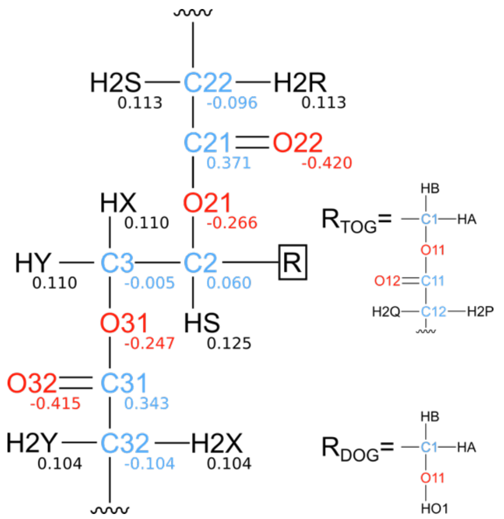

Figure S1. C36-p fine-tuned set of charges for TOG/DOG. For TOG, the charges of atoms in chain 1 are identical to those shown for chain 3.

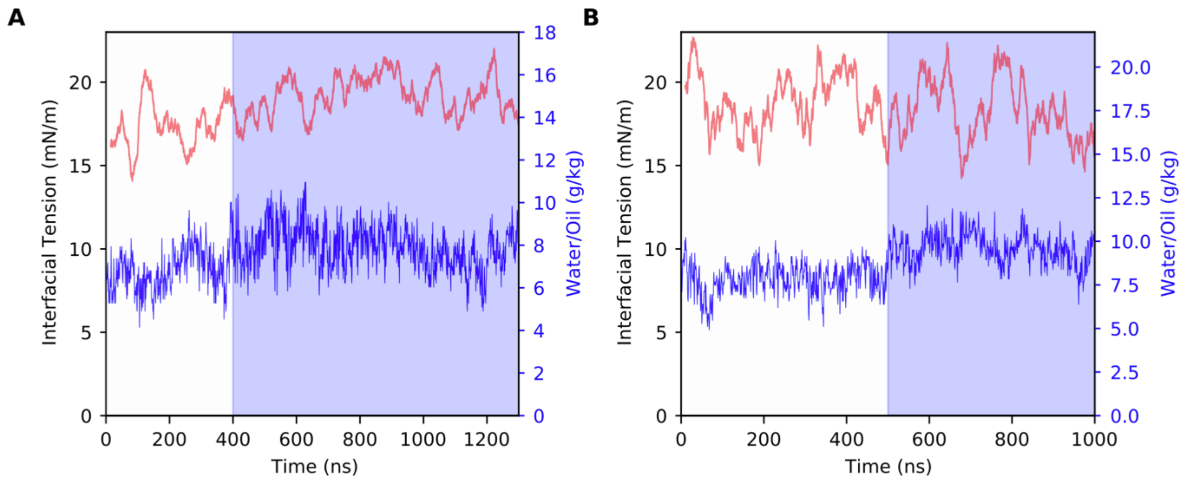

Figure S2. Interfacial tension (red) and water content (blue) of DOG computed from simulations carried out with the C36-c and C36-p models. (A) Results from the C36-c model. (B) Results from the C36-p model. The blue shaded region contains the part of the trajectory used to calculate the mean IT value for DOG, when water content shows convergence.

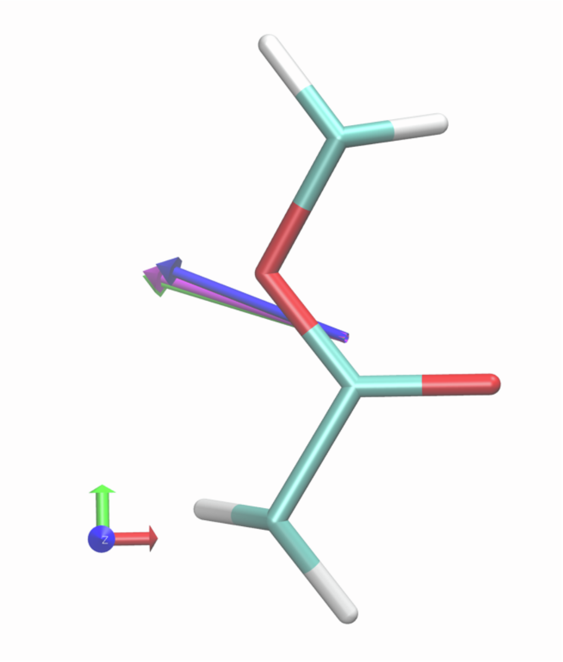

Figure S3. **Dipole moments orientation.** Dipole moments computed for a structure extracted from the glycerol-ester conformational ensemble by using the C36-s (in blue), C36-c (in green), and C36-p (in magenta) sets of charges, respectively. The origin of the dipole moment vectors was placed at the center of mass of the structure and pointing towards the center of the positive charge distribution (i.e., according to the physicist's convention).
